# Correction: Appropriate data segmentation improves speech encoding models: Analysis and simulation of electrophysiological recordings

**DOI:** 10.1371/journal.pone.0347704

**Published:** 2026-04-17

**Authors:** Ole Bialas, Edmund C. Lalor

The captions for Figs 1–4 are missing from the article. The captions have been provided here:

**Fig 1:**
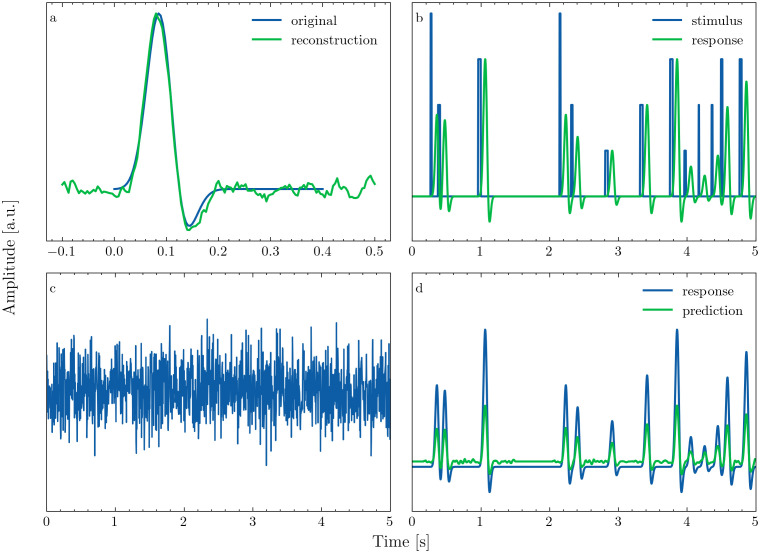
Generative simulation framework. a: Original wavelet used for generating and its reconstruction from the noisy response. b: Randomly generated stimulus sequence and simulated response obtained by convolving stimulus and wavelet. c: Simulated response with Gaussian noise added. d: Response predicted using the reconstructed TRF and original response, prior to adding noise. On all plots, the x-axis shows time in seconds and the y-axis amplitude in arbitrary units (a.u.).

**Fig 2:**
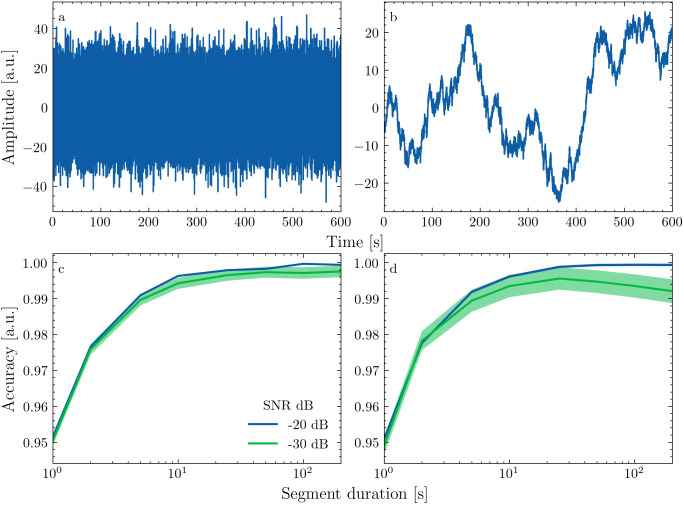
Simulation results. a and b show samples of Gaussian and 1/f noise with amplitudes in arbitrary units (a.u.). c and d show normalized reconstruction accuracy as a function of data segment length for two different SNRs.

**Fig 3:**
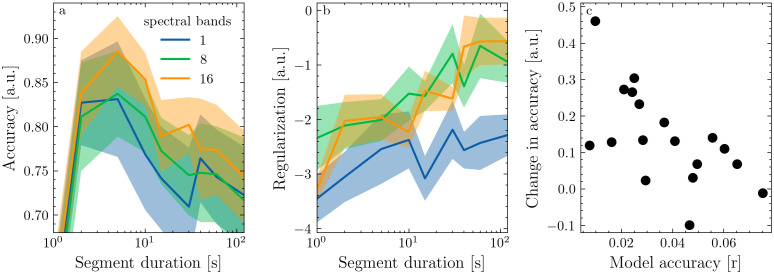
Effect of data segmentation on models for EEG responses to speech. a: normalized prediction accuracy in arbitrary units (a.u.) a function of segment duration for three models differing in spectral detail. b: optimal (log-transformed) λ decreases with segment duration. c: Relative difference in prediction accuracy between models fit on 120s and 10s segments for all participants as a function of their respective prediction accuracy. Please note that, while the y-axis in both a and c represents accuracy, these are different estimates that can not be compared directly.

**Fig 4:**
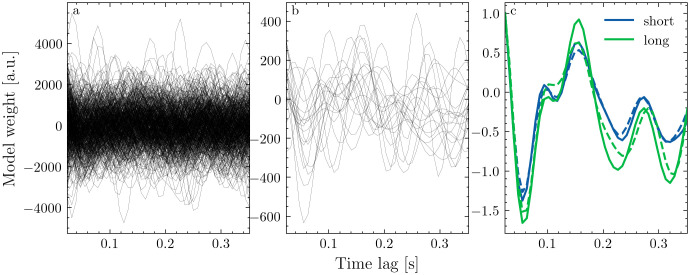
Segmentation regularizes the effect of outliers. a&b: TRF for each segment at a fronto-central channel after dividing the data into 5s and 120s segments, respectively with amplitude is arbitrary units (a.u.). c: Average TRF across all segments before (solid) and after (dashed) removing five percent of outliers.
