# Supplementary material for: Appropriate data segmentation improves speech encoding models: Analysis and simulation of electrophysiological recordings
Source: PLoS One. 2025 May 23;20(5):e0323276. doi: 10.1371/journal.pone.0323276 (PMC12101633; doi:10.1371/journal.pone.0323276)
Supplement: Attachment — Submitted filename: PONE-D-24-38627_comments.pdf [file pone.0323276.s001.pdf]

Line-by-line comments

30–31: While read speech may approximate "real speech," it still lacks many features of truly spontaneous speech. To substantiate this claim, consider citing the following studies:

> Howell, P., & Kadi-Hanifi (1991). Comparison of prosodic properties betweewn read and spontaneous speech material. *Speech Communication, 10*(2), 163–169. https://doi.org/10.1016/0167-6393(91)90039-V
>
> Mehta, G., & Cutler, A. (1988). Detection of target phonemes in spontaneous and read speech. *Language and Speech, 31*(Pt 2), 135–156. https://doi.org/10.1177/002383098803100203

47–49: Please clarify the definition of "estimand" to assist readers who may be unfamiliar with the term.

121–122: Upon reviewing Di Liberto et al. (2015) and Broderick et al. (2018), neither article specifies the actual material read by the speaker. If the original text is unavailable, could details regarding the narrative style or genre be provided? Understanding the type of text used could impact the interpretation of results.

Subject selection: 124–125

- What criteria were used for selecting the 19 participants in this study?
- Why was this particular EEG database chosen for the analysis?

131–136: The inclusion of the number of spectral bands as a variable is a valuable addition; however, they are infrequently referenced in the results and discussion sections. It would be beneficial to discuss their impact on the findings.

Modeling EEG responses: 143–145

- Please clarify the source of the "50 minutes" of data, as the analysis seems to involve only 19 subjects, each contributing between 170 and 200 seconds of data.
- What was the rationale for choosing these specific segment lengths? Given that Gonen and Tcheslavski (2012) identified a critical duration of 2 to 25 seconds, why not test segments up to 25 seconds using the current dataset?
- An exclusion criterion of 0.01 for model accuracy appears unusually low. Please provide justification for this threshold.

191–193: "On average" is stated twice in this sentence.

198: It might be beyond the scope of this paper, but was any post hoc analysis conducted to determine the factors driving variability across subjects?

202–204: The phrase, "What's more, there was a negative relationship (linear regression, $r = -0.62$, $p = 0.006$) between the relative change in accuracy due to segmentation…" needs clarification. Specifically, which segmentation is referenced here: the average of 10 and 120 seconds, the average across all segment durations, or the optimal segmentation for each subject?

206–208: Is there any commentary regarding the identified outlier?

209: Consider using "improve" or "increase" instead of "affect" to more powerfully convey the direction of the change.

Figures

- Although it is common in the field, including a definition for “a.u.” would be helpful for readers.
- Please correct the x-axis label from “segmend” to “segment.”
- **Figure 3a** & **c**: Although both y-axes are represented in a.u., are they on the same scale? For instance, does a 0.1 change in accuracy in panel **c** correspond to a 0.1 change in accuracy in panel **a**? If so, it would be helpful to specify this in the figure legend.
- **Figure 3c**: Regarding the “Change in accuracy,” which segmentation level was used to obtain these values?

While the Ethics statement is appreciated, it is somewhat confusing as neither author is affiliated with Trinity College Dublin. It would be helpful to include a sentence clarifying that the EEG data originated from Di Liberto et al. (2015) or Broderick et al. (2018) at that institution, allowing readers to understand the context without needing to consult those studies.
