# Supplementary material for: Appropriate data segmentation improves speech encoding models: Analysis and simulation of electrophysiological recordings
Source: PLoS One. 2025 May 23;20(5):e0323276. doi: 10.1371/journal.pone.0323276 (PMC12101633; doi:10.1371/journal.pone.0323276)
Supplement: Attachment — Submitted filename: rebuttal2.pdf [file pone.0323276.s003.pdf]

March 12, 2025

Dear Reviewer 1,

Thank you for your comments. In your response you requested:
”For unfamiliar readers, could the authorship or a brief description of the story be added?”
We have added two sentences to the section ”Modeling EEG responses to speech” that describe the content and tone of the narration to allow the reader to assess the emotional impact.

Sincerely,

Ole Bialas and Edmund Lalor
