# Supplementary material for: Appropriate data segmentation improves speech encoding models: Analysis and simulation of electrophysiological recordings
Source: PLoS One. 2025 May 23;20(5):e0323276. doi: 10.1371/journal.pone.0323276 (PMC12101633; doi:10.1371/journal.pone.0323276)
Supplement: Attachment — Submitted filename: rebuttal3.pdf [file pone.0323276.s004.pdf]

March 25, 2025

Dear Dr. Yang,

Thank you for your comments. We have added a summary to the title, it now reads:
Appropriate data segmentation improves speech encoding models: Analysis and simulation of electrophysiological recordings. We also replaced ”Introduction” with ”Background” in the structured abstract and put each point on a separate paragraph. In your last point, you requested that we add a table to our publication to make it easier for the reader to follow the main findings.
We believe that adding a table that compares the accuracy of the different encoding models with the different segment durations would likely be confusing and detracting rather than beneficial to readers. That is because the key point is the qualitative change in model accuracy that results from a change in segment duration, not the absolute values. We believe that the figures, combined with the text, illustrate this point well.

Sincerely,

Ole Bialas and Edmund Lalor
