# Supplementary material for: Appropriate data segmentation improves speech encoding models: Analysis and simulation of electrophysiological recordings
Source: PLoS One. 2025 May 23;20(5):e0323276. doi: 10.1371/journal.pone.0323276 (PMC12101633; doi:10.1371/journal.pone.0323276)
Supplement: Attachment — Submitted filename: rebuttal4.pdf [file pone.0323276.s005.pdf]

April 1, 2025

Dear Dr. Yang,

Thank you for providing an example of the desired formatting. We had inserted new paragraphs in the revised abstract, but the PLOS Latex template does not include a blank line after a paragraph. We changed the formatting of the abstract by manually inserting a blank line after every point. We also followed your suggestion to merge the ”Objectives” into the ”Background” section. We also made some minor changes for conciseness and hope that this meets the standards for publication.

Sincerely,

Ole Bialas and Edmund Lalor
